# Evolution of Wikipedia’s medical content: past, present and future

**DOI:** 10.1136/jech-2016-208601

**Published:** 2017-08-28

**Authors:** Thomas Shafee, Gwinyai Masukume, Lisa Kipersztok, Diptanshu Das, Mikael Häggström, James Heilman

**Affiliations:** 1 Department of Biochemistry and Genetics, La Trobe Institute for Molecular Science, Melbourne, Australia; 2 Department of Obstetrics and Gynaecology, University College Cork, The Irish Centre for Fetal and Neonatal Translational Research, Cork, Ireland; 3 Gravida: National Centre for Growth and Development, University of Auckland, Auckland, New Zealand; 4 Division of Epidemiology and Biostatistics, University of the Witwatersrand, School of Public Health, Johannesburg, South Africa; 5 Department of Family Medicine, Oregon Health & Science University, Portland, Oregon, USA; 6 Department of Paediatric Neurology, Universita degli Studi di Roma Tor Vergata, Roma, Italy; 7 Department of Paediatrics, Kothari Medical Centre and Research Institute, Kolkata, India; 8 Department of Pediatrics, ICARE Institute of Medical Sciences and Research, Haldia, India; 9 Working Group, Open Access India, India; 10 Department of Radiology, NU Hospital Group, Trollhättan, Sweden; 11 Department of Emergency Medicine, University of British Columbia, Vancouver, Canada

**Keywords:** health promotion, health education sa, inequalities, access to hlth care

## Abstract

As one of the most commonly read online sources of medical information, Wikipedia is an influential public health platform. Its medical content, community, collaborations and challenges have been evolving since its creation in 2001, and engagement by the medical community is vital for ensuring its accuracy and completeness. Both the encyclopaedia’s internal metrics as well as external assessments of its quality indicate that its articles are highly variable, but improving. Although content can be edited by anyone, medical articles are primarily written by a core group of medical professionals. Diverse collaborative ventures have enhanced medical article quality and reach, and opportunities for partnerships are more available than ever. Nevertheless, Wikipedia’s medical content and community still face significant challenges, and a socioecological model is used to structure specific recommendations. We propose that the medical community should prioritise the accuracy of biomedical information in the world’s most consulted encyclopaedia.

## Introduction

### Why should medical professionals care about Wikipedia?

Wikipedia is one of the most commonly read online sources of medical information, and is consistently among the top 10 most visited websites in the world (currently fifth).^1^ As well as being widely read by the general public, it is also used as a source of healthcare information by 50%–70% of physicians^2^ and over 90% of medical students.^3^ It is additionally used by educators, policymakers and journalists.^4–6^ Since the public relies on free online medical information for making health decisions, the accuracy and coverage of Wikipedia’s medical information have an immediate real-world impact on public health.^7^ The medical community should therefore take responsibility for ensuring its accuracy as an influential health information platform.

### Some background on the encyclopaedia and its relatives

**Figure 1:**
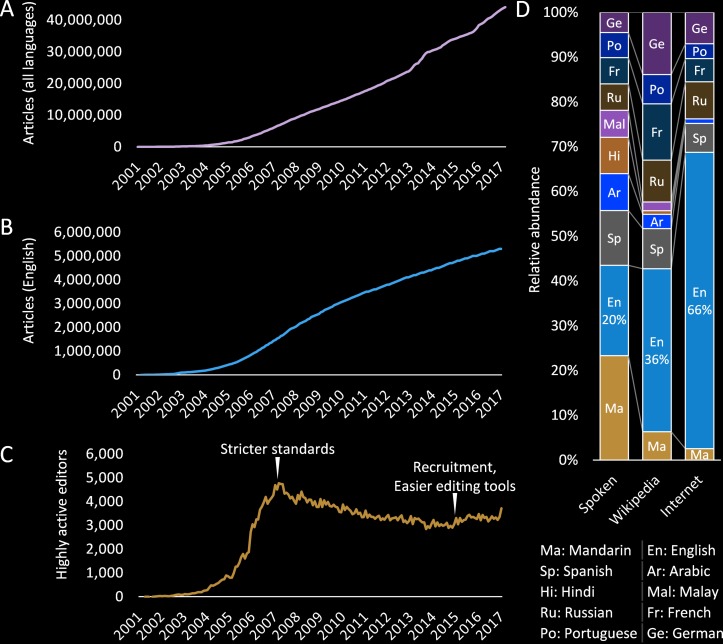
Wikipedia total size and editors. (A) Total number of articles in the encyclopaedia (all languages). (B) Total number of articles in the encyclopaedia (in English). (C) Total number of editors making >100 edits per month (in English). (D) The proportion of the top 10 most spoken languages (native + secondary) compared with the size of different language versions of Wikipedia, and usage on the internet as a whole.

Wikipedia is a massive online encyclopaedia with global reach and recognition.^8 9^ Its total content has grown rapidly since its inception in 2001, with 44 million articles across 295 languages, including >5.4 million in English as of May 2017 (figure 1A,B). The English-language Wikipedia is the largest and best known project supported by the Wikimedia Foundation (WMF), and will be the main focus of this article. Other projects host open-access images, education materials and structured data.^10 11^

After initial exponential growth of key topics, the English Wikipedia has settled into a slower, linear growth, as more niche topics and current affairs are added. These articles are written and edited by a community of approximately 30 000 editors that make >5 edits per month, and 3000 that make >100 edits per month. This number is down from its peak in 2007, when stricter content guidelines were introduced, but has remained stable over recent years, with a minor increase as easier writing and editing and tools are introduced (figure 1C). The size of the different language versions of Wikipedia is skewed towards English, although less in proportion to the internet as a whole (figure 1D).

Common criticisms of Wikipedia include concerns over content quality, coverage, readability and vandalism. However, much has been done to make Wikipedia’s open editing system remarkably robust—from editor culture and policies (eg, increased focus on reliable references)^12 13^ to technological improvements (eg, automated software that reverts vandalism).^14^ This has been reflected by improvements in perceived accuracy by readers.^15–17^ As the encyclopaedia’s contents, editors and policies change over time, studies of it can quickly go out of date. This article therefore aims to give an overview of the past, present and possible future of Wikipedia’s medical content.

## Current state of medical information on Wikipedia

### Content

**Figure 2:**
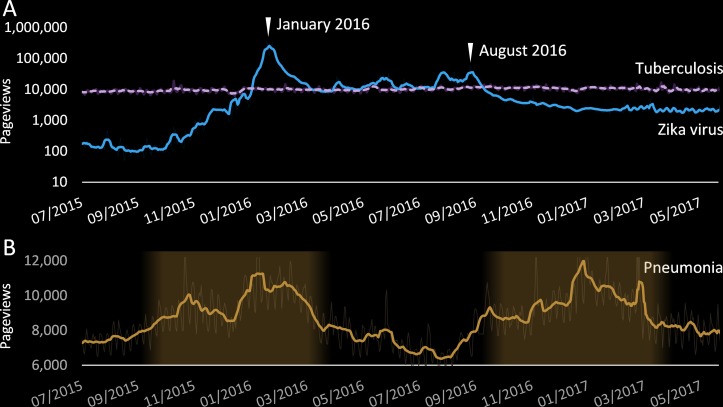
Daily pageviews for medical topics. (A) Most pageviews are relatively stable (eg, tuberculosis), while some are highly dependent on world events (eg, Zika virus). (B) Some seasonal illnesses vary cyclically (eg, pneumonia) by the seasons of the Northern Hemisphere, where approximately 90% of people live. All points smoothed by a 7-day moving average.

As of March 2017 there are 30 000 articles on medical topics in English Wikipedia, and another 164 000 in other languages. They are collectively read >10 million times per day.^1^ This extreme readership is only approximated by a few other resources, including the US National Institutes of Health and WebMD.^1^ Individual medical articles can often have thousands of views per day, although reader numbers for some are strongly affected by news coverage (eg, Zika virus; figure 2A) or seasonal (eg, pneumonia; figure 2B).^18 19^

**Figure 3:**
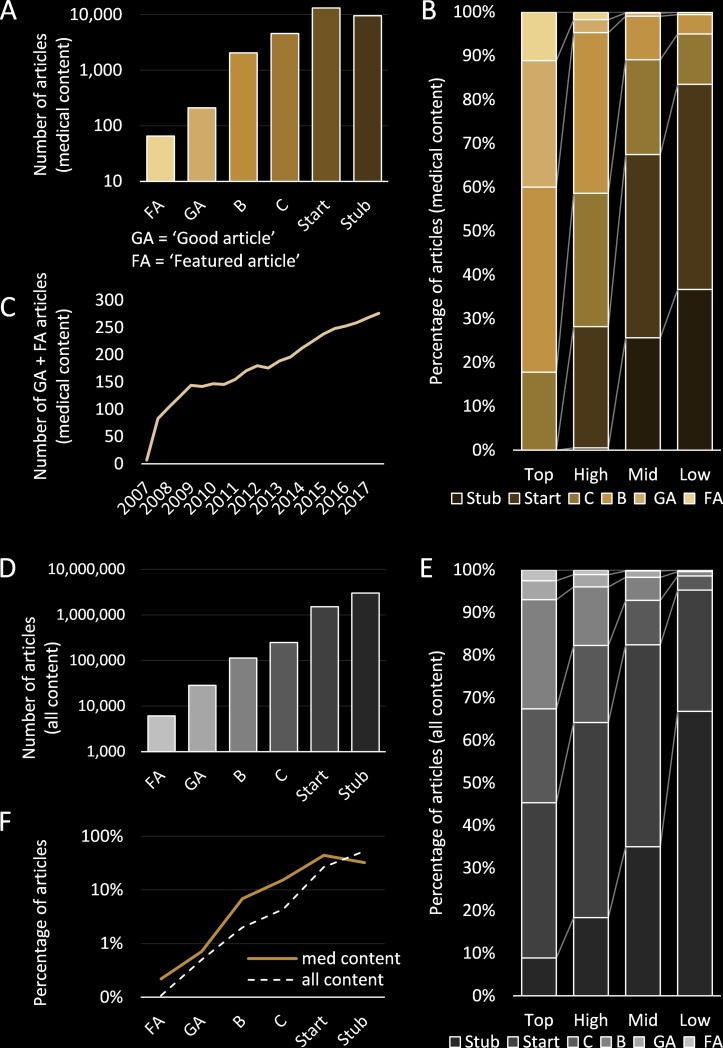
A summary of article quality across all articles in Wikipedia. Articles are assigned quality and importance rankings. ‘Featured article’ (FA) and ‘Good article’ (GA) are assigned by internal peer review. (A) Total number of medical articles of each quality ranking. (B) Percentage of medical articles at each quality ranking, separated by article importance (excluding unassessed). (C) The total number of medical articles (FA+GA) over time. (D) Overall number of all articles of each quality ranking Wikipedia-wide. (E) Overall percentage of all articles at each quality ranking, separated by articles importance (excluding unassessed) Wikipedia-wide. (F) A comparison between the overall rankings (dashed line) and those of medical articles (blue line). B, ‘B-class article’; C, ‘C-class article’.

Wikipedia articles are rated by importance and quality by the communities of editors (online supplementary tables S1 and S2). Top-importance articles include conditions of global significance, such as tuberculosis and pneumonia. High-importance includes common diseases and treatments. Mid-importance encompasses conditions, tests, drugs, anatomy and symptoms. The remaining low-importance articles include niche or peripheral medical topics such as laws, physicians and rare conditions. Articles are similarly rated for quality on a scale ‘Stub’, ‘Start’, ‘C’, ‘B’, ‘Good Article’ (GA) and ‘Featured Article’ (FA). The latter two categories are only assigned after an internal peer review process.^20^ GAs comprise 0.7% of medical articles and require a single peer reviewer (figure 3A,B). FAs comprise 0.2% of medical articles and have to pass more stringent criteria and often have 5–10 reviewers.

10.1136/jech-2016-208601.supp1 Supplementary file 1

The quality ratings of medical articles are well above Wikipedia’s average (figure 3D–F). In particular, 83% of the top-importance medical articles are of ‘B-class’ quality or above (only 30% Wikipedia-wide) and <1% of the top-importance and high-importance articles remain ‘Stub-class’ (25% Wikipedia-wide) (figure 3B,E). Over 270 medical articles have been promoted to GA and FA, with around 20 more passing review each year (figure 3C).

External assessment of Wikipedia’s overall content quality was found to be comparable to Encyclopaedia Britannica over a decade ago.^21^ Comparisons of its medical content with other sources vary for specific subjects, such as pharmacology, psychology or oncology^22–24^; however, some general conclusions can be drawn. Wikipedia’s medical content frequently suffers from low readability and errors of omission, despite the fact that included content is relatively high quality and well referenced.^12^ Errors are typically not due to deliberate vandalisation or underqualified editors,^25^ but rather that the volunteer editor base is relatively small and so topics are unevenly covered.

**Figure 4:**
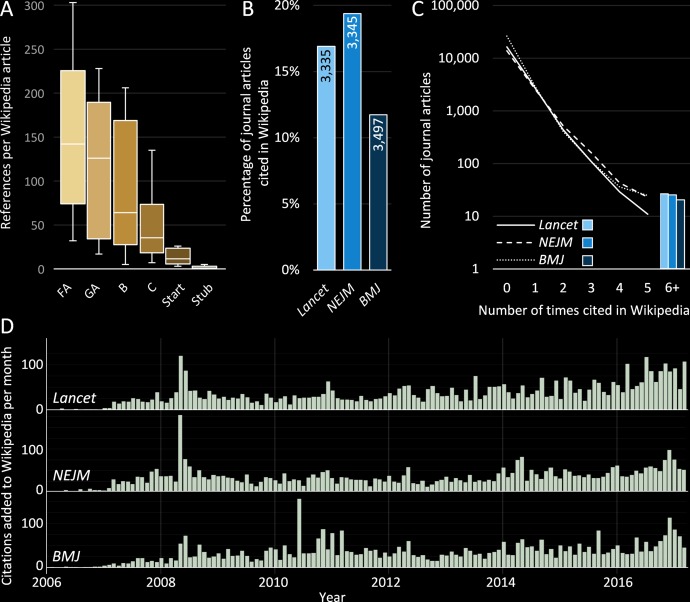
Citation metrics for medical content. (A) Total number of external references per Wikipedia medical article for different article qualities (n=10). FA, ‘Featured article’; GA, ‘Good article’; B, ‘B-class article’; C, ‘C-class article’. (B) Absolute numbers and percentage of scholarly articles from three representative high-impact medical journals that have been cited in Wikipedia (up to 13 January 2017). (C) Number of times articles from each representative medical journal have been cited in Wikipedia. (D) Dates when articles are first cited by Wikipedia from selected journals. *Lancet, The Lancet; NEJM, New England Journal of Medicine; BMJ, British Medical Journal*.

Improved referencing for Wikipedia’s medical articles has been a strong focus since 2007.^26^ Higher quality articles often cite more than a hundred references (figure 4). The majority of references for Wikipedia’s medical articles are drawn from reliable sources.^22 27^ Furthermore, secondary and tertiary sources (eg, meta-analyses and clinical guidelines) are strongly preferred in order to reflect the accepted medical consensus.^28^ Examples from three leading medical journals (*The Lancet*, *New England Journal of Medicine* and *British Medical Journal*) show similar trends, with a high percentage of articles cited by at least one Wikipedia article, and a subset of publications cited multiple times (following a power law).

In general, there is an upward trend in Wikipedia’s accuracy and reputation, but completeness and readability are still major limitations.^15–17^

### Community

Wikipedia editor communities are organised into approximately 800 currently active ‘WikiProjects’, which bring together editors interested in a particular topic or process in Wikipedia (online supplementary table S3).^29^ WikiProject Medicine was one of the first such communities, being founded in 2004 by Jacob de Wolff, MD. It is also one of the most active (consistently in the top 10, online supplementary figure S1) with 130 participants on its discussion forum in any given 90-day window, and a further 700 contributors who edit articles within its scope.^30^ The community’s overall size has remained relatively constant since 2013. These are largely a mixture of health professionals, researchers and students with an interest in freely available, accurate medical information.^25^ Discussion of improvements to content is typically held both at the WikiProject’s central discussion page^30^ and on the discussion pages for individual articles.^31^ The community has since expanded to form the Wiki Project Med Foundation, in 2012, a non-profit corporation working to promote the broader development and distribution of Wikipedia-related medical content.

### Collaborations

**Figure 5:**
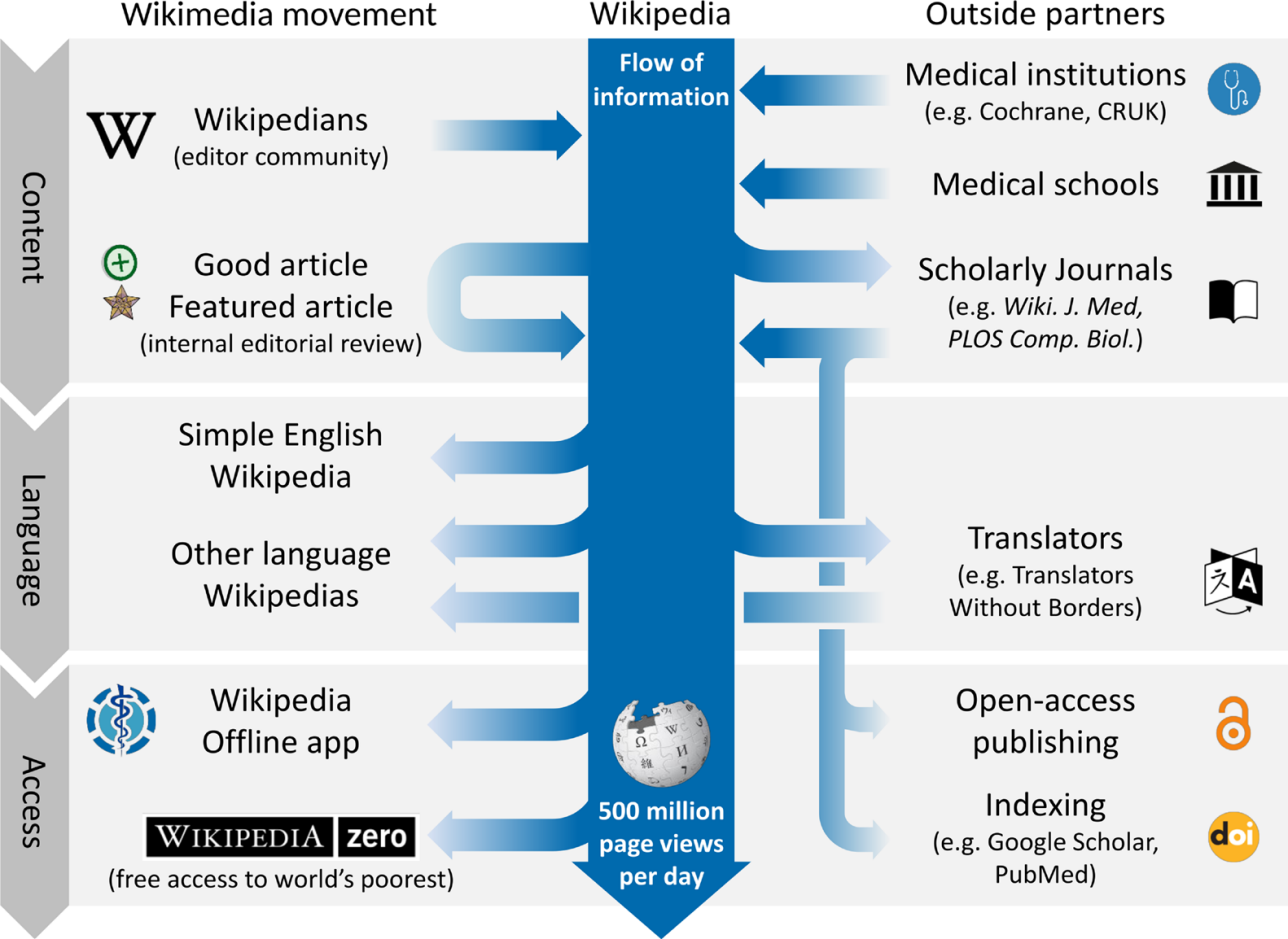
Collaborations between Wikipedia’s medical community and outside partners. An outline of collaborations and interactions between the WikiProject Medicine community, institutions, schools, journals and translators (adapted from reference Shafee et al).^32^ CRUK, Cancer Research UK; *PLOS Comp Biol*, *PLOS Computation Biology*; *Wiki J Med*, *WikiJournal of Medicine*.

One of the largest changes in Wikipedia over recent years has been an increasing number of collaborations between the encyclopaedia and the wider biomedical community (figure 5).^32^ Dozens of academic publishers, medical institutes and universities have formed temporary and extended partnerships. These benefit the encyclopaedia by providing and improving content, and benefit the partner organisations by increasing impact and awareness as a result of Wikipedia’s readership.

One such example is Cochrane, which performs systematic reviews of healthcare research. Since 2012, the organisation has been collaborating with editors to keep articles accurate, up-to-date and evidence-based, and has recruited a ‘Wikipedian in residence’ to advise and coordinate efforts. For example a 4-month project at the end of 2016 with a team of medical students updated over 100 pages.^33^ Cancer Research UK has similarly added hundreds of diagrams and animations.^34^ Multiple medical schools have experimented with organising students to edit Wikipedia articles, teaching writing and referencing skills, as well as the value of open access to information.^35^

One of the longest standing biomedical projects has been the ‘Gene Wiki’ initiative to create Wikipedia articles on all human genes. It began in 2008 with the automated creation of a stub article for every human gene.^36^ Initially, 7500 articles were created and 650 updated, with that total since rising above 11 000 as new stubs are automatically added.^36^ In 2012 the project formed a further collaboration with the journal *Gene*, whereby articles can be submitted to the journal, then adapted to update the relevant Wikipedia page. This has led to 62 publications integrated into >90 Wikipedia articles.^37^ Furthering this precedent, the journal *RNA Biology* requires that new RNA families be added to Wikipedia when published in the journal.^38^ A more direct dual-publishing format has been developed by the Public Library of Science (PLOS).^39 40^ ‘Topic Page’ review articles are published in either *PLOS Computational Biology* or *PLOS Genetics*, and then published into Wikipedia to seed a new page, producing 11 such articles since 2014. This model is extended by the *WikiJournal of Medicine*, an open-access medical journal hosted by the WMF, which specialises in this type of integrated publishing.^41^ It has additionally put existing Wikipedia articles through academic peer review,^41^ following the first experiment in doing so by *Open Medicine* in 2014.^42^

Translation and distribution collaborations increase the impact of content improvement efforts. Translators without Borders, a non-profit organisation, collaborates to translate important medical articles on Wikipedia for the non-English Wikipedias, so far resulting in more than 5.3 million words of translated text in over 100 languages.^43^ Several telecom operators in Africa, South East Asia and the Middle East waive data fees for Wikipedia access (Wikipedia Zero), and free mobile apps allow offline storage of Wikipedia medical content for those without reliable internet access.^44^ The success of such ventures is highlighted by the 2014 Ebola outbreak. During the early part of the outbreak, teams overhauled the English-language articles on Ebola, and translated them into more than 100 languages. This content was viewed at least 89 million times in 2014 (at a conservative estimate) and was likely the most used online source for Ebola information in each of the four most affected countries.

Overall, these diverse initiatives add and disseminate high-quality content, as well as introduce Wikipedia editing to people who otherwise would not have contributed.

## Future of medical content on Wikipedia

### Challenges

Given Wikipedia’s importance as a source of information, it is crucial that it is continually improved and updated. Despite its successes, significant challenges remain to be overcome (table 1). Errors and omissions need to be reduced and language often needs to be simplified.^23 45^ Content is still heavily skewed towards English. Editor numbers are still insufficient to support expertise on the diversity of specialist topics, and a wider demographic of contributors needs to be recruited.^46^ The encyclopaedia norms and bureaucracy need to be simpler and clearer to reduce negative experiences for new editors and to better interface with partner organisations.^47^ In general, the rewards for contributing need to justify the time commitment for expert contributors.

**Table 1:** Summary of some of the main challenges facing Wikipedia and example measures to address them

Challenge	Proposed solution(s)
**Content accuracy, readability and language bias**^23 45 60^ Highly variable article quality Much important information only available to English-language speakers	Greater participation of experts—edits beget edits^48^ Content addition through external partnerships Automated language complexity information Tools to recommend articles for translation^60^
**Editor numbers, demographic bias and expertise**^46 56^ Size of the community insufficient to support sufficiently diverse expertise Over-representation of male, white editors from high-income countries Tracking and reward for contribution still underdeveloped	Target diverse editor recruitment Support interface and cultural changes that reduce disadvantage for under-represented groups^46^ Develop more sophisticated ways to summarise a contributor’s impact^56^
**Bureaucracy and policy complexity**^47^ Wikipedia’s norms and policies can be different from those that medical and research professionals are used to, and their descriptions are overly complex.	Simplify and consolidate rules, particularly for new contributors Promote compatible collaboration models with external partners

The initiatives described in the previous section show how engagement by other organisations can bolster the efforts of Wikipedia’s established community of medical content editors. Improved content also generates a positive feedback cycle of increased editing.^48^ The WMF is currently developing a strategic plan for the coming 15 years for Wikipedia and its sister projects.^49^ In this section, we therefore describe recommendations for established Wikipedia contributors, as well as the medical, research and publishing communities.

Here we use a socioecological framework to make recommendations for the encyclopaedia’s main individual, societal, physical and organisational challenges.^50^ We suggest an emphasis on collaboration between Wikipedia and external partners, strategies to ensure content quality, better access for low-income and middle-income countries, and improved training and outreach.^49^

### Individual

The backbone of Wikipedia is the individual contributions of volunteers. Individual attitudes, behaviours and knowledge consequently have a strong impact.

Wikipedia suffers from many of the same issues in representing the global population as the Science, Technology, Engineering and Mathematics community as a whole.^46^ For example, only 10%–20% of editors identify as female.^46^ Countering systemic bias towards over-representation of Northern Hemisphere white men will require active recruitment and engagement efforts towards under-represented groups.^51 52^ Supporting that recruitment will also require ensuring that the editing interface and culture do not disadvantage under-represented groups.^46^ Simultaneously, encouraging engagement by expert contributors is clearly beneficial for ensuring content accuracy.^36 48^ Medical and research professionals are busy, and for Wikipedia to be prioritised it is necessary to reiterate Wikipedia’s key role as an outreach and public health platform.^44^ This includes individually engaging established professionals, as well as training medical and other health sciences students in editing.^34^

The Wikipedia editing community has also had a reputation for being antagonistic and intolerant of mistakes.^53^ We strongly endorse the ongoing work by the editor community and WMF to improve community inclusivity.^53 54^ It is also worth noting that, when trained to edit Wikipedia, students are remarkably robust to criticism of their errors, highlighting the value of improved tutorials, introductory material and mentoring.^55^

### Societal

Wikipedia exists within a broad societal context of multiple overlapping communities, institutions and cultures. Although it is widely used, opinion of Wikipedia in academic circles has often been negative. The consideration of Wikipedia as a low-quality source of evidence discourages contributions and removes the positive reinforcement of recognition for this work.^48^ This problem is diminishing as content quality increases and consequently the reputation of Wikipedia improves.^15–17^ Rewarding contributors by tracking and recognising their impact would have a large effect on expert contributors.^56^

Tracking and summarising Wikipedia contribution are still difficult. Editors can associate their accounts with their Open Researcher and Contributor ID or Reuters ResearcherID. However, Wikipedia contribution is typically quite different from other forms of authorship. Some Wikipedia articles are written by a small number of authors, like a traditional publication, but it is also common for editors to contribute a small amount to a very large number of articles. New standardised metrics are needed to describe the varied work done in writing, reviewing, improving, debating and illustrating Wikipedia’s content, as well as its impact.^56–58^

Medical organisations are already adapting to recognise the diverse ways in which members contribute to public health. The increasingly common Altmetric score includes Wikipedia as one of the indicators of societal impact for academic publications.^59^ Recognition and support for Wikipedia content contributions will similarly impact whether experts are able to prioritise engagement in improving Wikipedia.

### Physical

Simplifying Wikipedia interfaces can increase article generation by reducing barriers to the technical requirements of contributing to an online encyclopaedia. Reducing complexity and increasing automation of common tasks allow editors to concentrate on content. Additionally, consolidating rules and guidelines will lower barriers for medical practitioners and academics to join or interact with the Wikipedia community. The editing technology, interface and workflow influence who is able to contribute and so influence editor diversity.^46^ For example, making it easier for interested editors to find articles that would benefit from translation will help reduce the English-language bias.^60^ Automatic feedback on readability of added content can similarly support improved writing. The increasing worldwide internet access, particularly on mobile devices, will further increase use of Wikipedia by academics and medical practitioners less experienced in the technological skills. Further development of tools for non-expert editors will enhance contributions and grow the editor community.

### Organisational

The collaborative efforts of medical institutions and Wikipedia are vital to support expert contributors.^44^ Wikipedia’s relatively extreme egalitarian, open-access and transparent systems can clash with the established norms of medical institutions. Wikipedia’s policies and guidelines have become increasingly complex and very different from those that new editors or partner organisations are accustomed to.^47^ For example, Wikipedia’s protections for anonymity are unusual to a profession where accountability and verified expertise are the norm. Partner organisations have to work out how to interact with Wikipedia systems. Conversely, the Wikipedia community needs to support this by consolidating its policies and guidelines. This will allow new users to avoid accidental errors while they learn the nuances of Wikipedia contribution, and help organisations work out compatible partnerships. Successful models already exist on how to achieve productive collaborations, as discussed in the previous section, and we posit that the public health outcomes are worth the effort.

## Conclusion

Wikipedia is set to retain its position as a key public health information source. Its content, community, collaboration and challenges have been constantly evolving since it was established in 2001. Proposed socioecological recommendations are most successful when compounded. Many of these issues also involve positive feedback effects; for example, better representation of female editors encourages more to join, and improvement of Wikipedia’s reputation encourages expert contribution. Now is a particular period of change as the WMF is currently soliciting feedback to help shape its strategic plan through to 2030. Opportunities for the medical community to shape the encyclopaedia’s future stem from individual engagement with its ‘anyone can edit’ model, and increasing partnerships with the wider medical ecosystem. The medical community must work together to ensure that medical content is accurate in the world’s most consulted encyclopaedia.

**What is already known on this subject**

- Wikipedia is one of the most used medical information resources globally, with immediate public health implications.
- Its model of user-generated content presents unique challenges and opportunities.
- Its content quality is variable but improving, and in need of further expert input.

**What this study adds**

- We summarise the major trends in how Wikipedia’s medical content, community and collaborations have changed since its inception in 2001.
- We raise specific proposals for both the Wikipedia community and medical institutions to help improve the encyclopaedia.
