# Supplementary material for: Evolution of Wikipedia’s medical content: past, present and future
Source: J Epidemiol Community Health. 2017 Aug 28;71(11):1122–9. doi: 10.1136/jech-2016-208601 (PMC5847101; doi:10.1136/jech-2016-208601)
Supplement: Supplementary file 1 [file jech-2016-208601supp001.pdf]

Supplementary information

The evolution of Wikipedia's medical content: past, present and future

Thomas Shafee <T.Shafee@latrobe.edu.au>, Gwinyai Masukume, Lisa Kipersztok, Diptanshu Das, Mikael Häggström, James Heilman

Figures and Tables 2

Supplementary Table S1 | Article ratings across medical articles in Wikipedia 2

Supplementary Table S2 | Article ratings across all articles in Wikipedia 2

Supplementary Table S3 | WikiProject editor communities 3

Supplementary Figure S1 | Wikiproject participation numbers 4

Methods 4

References 5

Figures and Tables

Quality	Importance					
	Top	High	Mid	Low	Unassessed	Total
FA	10	17	19	19		65
GA	26	31	68	86		211
B	40	352	871	785		2048
C	16	307	1944	2289		4556
Start		258	3753	9135	8	13154
Stub		6	2232	7356	5	9599
Unassessed					22	22
Total	92	971	8887	19670	35	29655

Supplementary Table S1 | Article ratings across medical articles in English Wikipedia
Number of articles in each importance and quality category. Ratings are set and updated by Wikipedia's medical editor community, WikiProject Medicine. FA='Featured article' and GA='Good article' are only assigned by internal peer review.

Quality	Importance					
	Top	High	Mid	Low	Unassessed	Total
FA	1,199	1,847	1,737	1,100	191	6,074
GA	2,119	4,847	9,477	10,348	1,757	28,548
B	12,222	23,130	35,423	28,494	13,956	113,225
C	10,488	30,487	68,122	94,937	44,033	248,067
Start	17,343	77,119	309,766	808,221	296,172	1,508,621
Stub	4,239	30,919	228,711	1,895,512	845,065	3,004,446
Unassessed	139	435	1,661	16,011	532,091	550,337
Total	51,190	181,248	691,196	2,951,044	1,795,628	5,670,306

Supplementary Table S2 | Article ratings across all articles in English Wikipedia
Number of articles in each importance and quality category. Ratings are set and updated by the relevant editor communities on Wikipedia. FA='Featured article' and GA='Good article' are only assigned by internal peer review.

	Articles	Participants	Editors
**Deletion sorting [a]**	n/a	211	n/a
**Women in Red [b]**	n/a	176	n/a
**Military history**	163795	151	3175
**Articles for creation [a]**	39886	142	723
**Football**	195831	127	2888
**Did you know [c]**	n/a	110	n/a
**Guild of Copy Editors [a]**	n/a	103	n/a
**Medicine**	28825	101	884
**Video games**	33351	92	1113
**Resource Exchange [a]**	n/a9	85	n/a
**Film**	136461	79	3065
**New pages patrol [a]**	n/a	73	n/a
**Biography**	1362159	47	16121
**Television**	80547	45	3267
**Mathematics**	14919	41	354

Supplementary Table S3 | WikiProject editor communities

The top 15 most active communities in English Wikipedia, as defined by their number of active participants (of 810 active wikiprojects with ≥1 participants). Participants have made ≥2 edits within 90 days to the project's discussion page. Editors have made ≥5 edits over 30 days to any article within the project's scope. Some projects do not have a permanent set of 'in scope' articles and so are indicated with 'n/a'. [a] 'Deletion sorting', 'Articles for Creation', 'New Page Patrol', 'The Guild of Copy Editors', and 'Resource Exchange' are general content quality groups. [B] 'Women in red' is a community dedicated to redressing systemic bias. [c] 'Did You Know' organises the facts that appear on the front page.

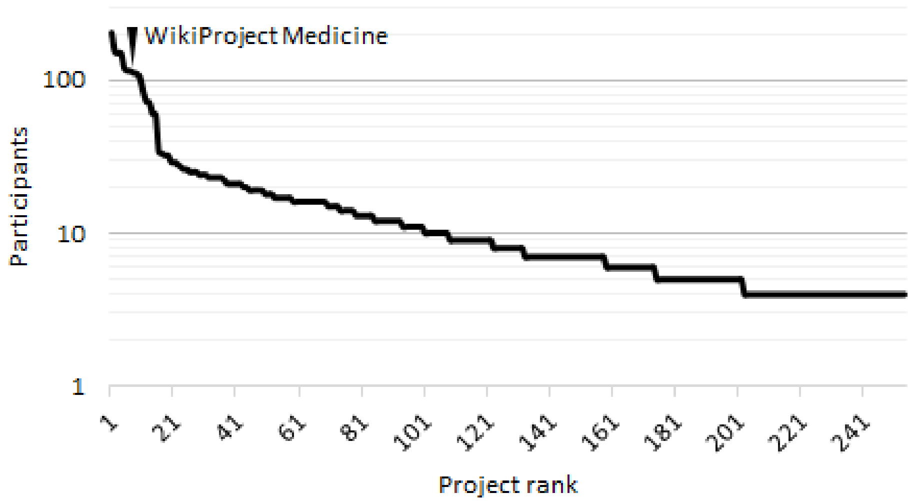

Supplementary Figure S1 | Wikiproject participation numbers

Participant number for the 254 WikiProjects with 4 or more participants (out of 810 with ≥1). Triangle indicates Wikiproject Medicine (rank=8, participants=101).

Methods

All data was downloaded from publically accessible databases for analysis during May 2017 unless otherwise mentioned. Data on Wikipedia article importance and quality assessments were downloaded from 'WP:MED' and 'WP:STATS' compiled by the WP 1.0 bot.[1–3] Historical logs of medical Featured and Good Articles were downloaded from the medical article assessment statistics history log (January and July from 2008-2017).[4] Article page-view statistics were downloaded from the WMFlab Pageviews tool, and page-views for the total medical content were downloaded from the WMFlab Massviews tool.[5,6] WikiProject statistics were downloaded from the WikiProject Directory and the history log compiled by Reports bot.[7–9] The 'Participants' and 'Editors' definitions are used by the directory and were developed by 'Wikiproject X' and the 'Wikiproject council' in 2015. The overall size and editor numbers were downloaded from Wikimedia Statistics and WikiProject Medicine Statistics.[10,11]

Data for Wikipedia citations of Lancet, New England Journal of Medicine, and British Medical Journal articles was downloaded from the AltMetric database using the Altmetric Explorer v2. Random articles were chosen using the *sample()* function in the [R] programming language. Statistics on publication of wikipedia pages was gathered from the relevant Wikipedia category and the WMFlab Massviews tool.[6,12] Gene Wiki Review statistics were gathered from the Gene Wiki Review virtual special issue and the WMFlab Template Linking tool.[13,14] Language data was downloaded from SIL International's 2015 Ethnologue database, the W3 2015 Web Technology Survey, and Wikimedia Statistics.[15–17] Description of the editing guidelines for medical content were based on WP:MEDRS.[18] Featured article reviewer data for 2016 provided by Mike Christie during February 2017.[19]

Viewership numbers for the Ebola-related content in 2014 was estimated from the 2014 readership data for medical articles collated by Andrew West. The sum views in English on Desktop were 24.4 million (Ebola virus disease 18.6M; Ebola virus 3.0M; Ebola virus epidemic in West Africa 2.7M; List of Ebola outbreaks 0.7M; Ebolavirus 0.4M).[20] This value was adjusted for mobile and non-english views as follows. In 2014, 41% pageviews were from mobile (for all of English Wikipedia) though medicine traditionally has a greater than average mobile viewership,[21] so total English Wikipedia readership on all platforms was approximately 41.4 million. In 2013, 53% of pageviews were to non-English language Wikipedias,[21] though again the Ebola articles existed in many more languages than the typical WP article (109 languages in 2014).[22] A highly conservative estimate is therefore 88.7 million views across all platforms and languages of Ebola-related content.

References

1. WikiProject Medicine (WP:MED). *Wikipedia* Available at: https://en.wikipedia.org/w/index.php?title=Wikipedia:WikiProject_Medicine&oldid=772497390. (Accessed: 25th May 2017)
2. User:WP 1.0 bot. *Wikipedia* Available at: https://en.wikipedia.org/w/index.php?title=User:WP_1.0_bot&oldid=705614927. (Accessed: 25th May 2017)
3. Statistics (WP:STATS). *Wikipedia* Available at: https://en.wikipedia.org/w/index.php?title=Wikipedia:Statistics&oldid=780909203. (Accessed: 26th May 2017)
4. Version 1.0 Editorial Team - Medicine articles by quality statistics Revision history. *Wikipedia* Available at: https://en.wikipedia.org/w/index.php?title=Wikipedia:Version_1.0_Editorial_Team/Medicine_articles_by_quality_statistics&action=history. (Accessed: 25th May 2017)
5. Pageviews Analysis. Available at: https://tools.wmflabs.org/pageviews. (Accessed: 25th May 2017)
6. Massviews Analysis. Available at: https://tools.wmflabs.org/massviews. (Accessed: 25th May 2017)
7. WikiProject Directory. *Wikipedia* Available at: https://en.wikipedia.org/w/index.php?title=Wikipedia:WikiProject_Directory/All&oldid=780839060. (Accessed: 25th May 2017)
8. WikiProject Directory Revision history. *Wikipedia* Available at: https://en.wikipedia.org/w/index.php?title=Wikipedia:WikiProject_Directory/All&action=history. (Accessed: 25th May 2017)
9. User:Reports bot. *Wikipedia* Available at: https://en.wikipedia.org/w/index.php?title=User:Reports_bot&oldid=782232654. (Accessed: 25th May 2017)
10. Wikipedia Statistics. *Wikipedia* Available at: https://stats.wikimedia.org/EN/TablesWikipediaEN.htm. (Accessed: 25th May 2017)
11. WikiProject Medicine Statistics. *Wikipedia* Available at: https://en.wikipedia.org/w/index.php?title=Wikipedia:WikiProject_Medicine/Stats/Number_of_articles_

by_language_2017&oldid=783111222. (Accessed: 25th May 2017)

12. Category:Wikipedia articles with sections published in WikiJournal of Medicine. *Wikipedia* Available at: https://en.wikipedia.org/w/index.php?title=Category:Wikipedia_articles_with_sections_published_in_WikiJournal_of_Medicine&oldid=770678863. (Accessed: 25th May 2017)
13. Gene Wiki Review virtual special issue. Available at: http://www.sciencedirect.com/science/journal/03781119/vsi. (Accessed: 25th May 2017)
14. Template linking and transclusion check. Available at: http://tools.wmflabs.org/templatetransclusioncheck/index.php?lang=en&name=Template%3AInfobox+gene. (Accessed: 25th May 2017)
15. Summary of worldwide language sizes. *Ethnologue* Available at: https://www.ethnologue.com/statistics/size. (Accessed: 25th May 2017)
16. Usage Statistics and Market Share of Content Languages for Websites, May 2017. Available at: https://w3techs.com/technologies/overview/content_language/all. (Accessed: 25th May 2017)
17. Wikipedia Statistics - Language sizes. Available at: https://stats.wikimedia.org/EN/Sitemap.htm. (Accessed: 25th May 2017)
18. Identifying reliable sources in medicine (WP:MEDRS). *Wikipedia* Available at: https://en.wikipedia.org/w/index.php?title=Wikipedia:Identifying_reliable_sources_(medicine)&oldid=778280427. (Accessed: 25th May 2017)
19. Christie, M. FAC nominations August 2016 through January 2017. *Wikipedia* Available at: https://en.wikipedia.org/w/index.php?title=User:Mike_Christie/Sandbox9&oldid=764296719. (Accessed: 25th May 2017)
20. West, A. 2014 Top Medical Articles. *Wikipedia* Available at: https://en.wikipedia.org/w/index.php?title=User:West.andrew.g/2014_Top_Medical_Articles&oldid=670253315. (Accessed: 25th May 2017)
21. Heilman, J. M. & West, A. G. Wikipedia and medicine: quantifying readership, editors, and the significance of natural language. *J. Med. Internet Res.* **17,** e62 (2015).
22. Ebola virus disease data. *WikiData* Available at: https://www.wikidata.org/w/index.php?title=Q51993&oldid=184768131#sitelinks-wikipedia. (Accessed: 25th May 2017)
